# Assessing the impact of implementing the clinical protocol and therapeutic guidelines in COPD in real-life

**DOI:** 10.1016/j.clinsp.2024.100529

**Published:** 2024-11-08

**Authors:** Daiana Abreu Lourenço Sales, Priscila Carla Moura Honório, Vanusa Barbosa Pinto, Frederico Leon Arrabal Fernandes, Regina Maria de Carvalho Pinto, Alberto Cukier

**Affiliations:** Hospital das Clínicas da Faculdade de Medicina da Universidade de São Paulo (HC-FMUSP), São Paulo, SP, Brazil

The Clinical Protocols and Therapeutic Guidelines (PCDT) are documents issued by the Brazilian Ministry of Health, which must be followed by managers of the Unified Health System (SUS). The PCDT for the management of Chronic Obstructive Pulmonary Disease (COPD) was published in 2021.^1^ Until the publication of the 2021 PCDT, the health department of the state of São Paulo dispensed long-acting inhaled anticholinergics through its own protocol.^2^^,^^3^

In the context of the state protocol in force until 2021, patients with severe or very severe COPD treated in our service, a tertiary university hospital, used long-acting anticholinergics, formoterol + budesonide or salmeterol + fluticasone, alone or in combination. With the new PCDT,^1^ these medications were replaced by dual bronchodilation combinations in a single device (tiotropium + olodaterol or umeclidinium + vilanterol) combined or not with inhaled corticosteroids in a separate device (budesonide or beclomethasone).

From the first consultation returns of patients treated according to PCDT 2021, we noticed dissatisfaction in many of them. We designed a research protocol with the aim of evaluating this perception (D.A.L.S. residency completion work in Pharmacy. Ethics review board approval number 71,628 323.3.0000.0068; 19/10/2023).

We report the impact of implementing the 2021 COPD PCDT[1] on 52 sequential patients from our outpatient clinic, interviewed between May and December 2023 using a structured questionnaire, applied as part of pharmaceutical care and administered after signing informed consent. The interviews were carried out at an average interval of 196 ± 87 days after changing the treatment. Of the patients evaluated, 33 (63.5 %) were female. Forty-three (82.7 %) were aged between 60 and 79 years old and were former smokers. Regarding lung function, the mean FEV1 was 37±14.7 %. Regarding the worsening of symptoms in the last 12 months, 27 (51.9 %) patients had 1 or more exacerbations and 7 (13.4 %) required hospitalization.

The research participants previously used tiotropium 2.5 mcg (73.1 %), generally associated with formoterol 12 mcg + budesonide 400 mcg (55.8 %) or salmeterol + fluticasone in a nebulimeter at a dose of 25/125 mcg (25 %) or powder 50/250 mcg (3.8 %). The implementation of the medication change took place in the second half of 2022.

Thirty-two patients (63 %) were switched to tiotropium 2.5 mcg + olodaterol 2.5 mcg and 20 (37 %) to umeclidinium 62.5 mcg + vilanterol 25 mcg. Forty-one were on triple therapy with beclomethasone 250 mcg (40 %) or budesonide 200 mcg (39 %).

At the time of evaluation, taking into account pulmonary symptoms and limitations in daily activities, 19 patients (36.5 %) reported that they had worsened after changing treatment, 17 (32.7 %) reported improvement and 14 (26.9 %) stated that they had not observed any changes with the change. In Fig. 1, it is possible to observe that the self-perception of symptom control is reflected in the participants' degree of satisfaction regarding the change in treatment. Fifteen patients who reported dissatisfaction reported the concomitant use of formoterol + budesonide (*n* = 9) or salmeterol + fluticasone (*n* = 6) to relieve symptoms.

**Fig. 1:**
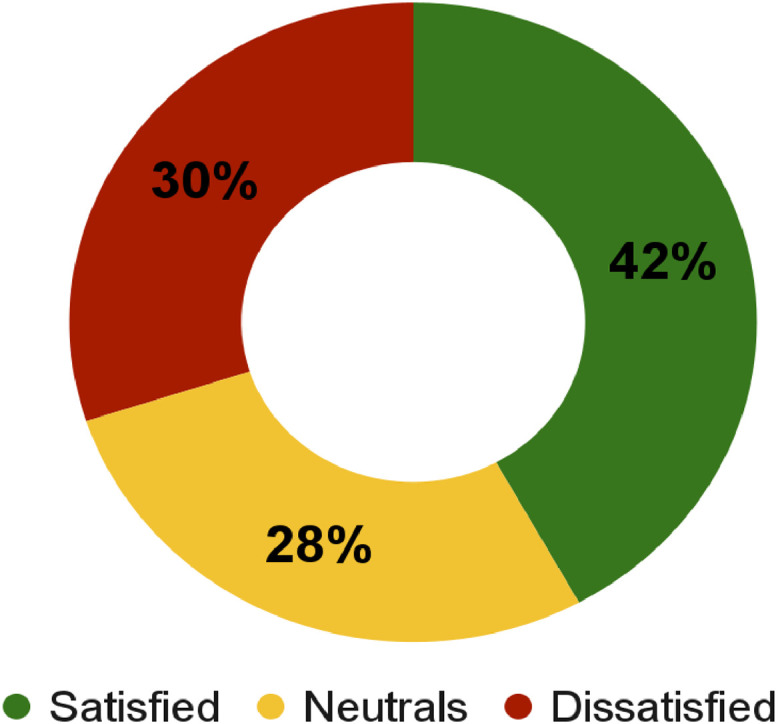
Participants' degree of satisfaction regarding the change in inhalation treatment.

In theory, the availability of double bronchodilation associated with the same device applied in a single daily dose should increase adherence to treatment. In practice, we observed that the majority of patients did not report improvement with this strategy. Why did approximately one-third of patients who were offered the best available medication option experience a worsening of their condition?

When analyzing the data, we identified some possibilities.

The simple fact of changing medication in a stable patient can lead to worsening control. All patients were forced to switch to new combinations, regardless of their current clinical condition. Many had their prescription modified and were stable and satisfied with the therapy used. A Swedish study^4^ reported that simply changing the inhaler device using the same active ingredient resulted in worsening control in asthmatics, leading to a greater number of exacerbations and unscheduled appointments. Usmani et al. concluded, in a recent systematic review,^5^ that the modification of inhaled medications in patients with asthma or COPD for non-clinical reasons is a complex issue, with variable clinical consequences, and may be harmful to the relationship between patients and healthcare professionals, especially when changes are not consented.

Another explanation lies in the circadian variability of symptoms. In a national multicenter study,^6^ a considerable portion reported morning symptoms and/or nighttime awakenings due to respiratory complaints. Reviewing the literature, the authors reported several studies in which morning symptoms were reported by 37 %‒81 % of patients and evening symptoms by 25 %‒68 %. It is not surprising, therefore, that some COPD patients value drugs with rapid onset of action twice a day.^7^^,^^8^

A third hypothesis lies in the initial difficulty of using inhalation devices in some patients. Although our outpatient clinic has a pharmaceutical care program in which the inhalation technique is reviewed, we know that patients require multiple instructions to achieve an adequate level of technique for using the device,^9^ revealed, for example, by the difficulty in using soft mist inhalers in 27 % of patients evaluated, despite being trained.

Our study has some limitations. We analyzed a small number of patients, from the most severe spectrum of the disease, in a single, specialized, university-level center with a multidisciplinary structure. It was a retrospective evaluation of a population with multiple variables such as inconsistent medication supply^10^ and comorbidities that could influence clinical control. Finally, our analysis was limited to short-term outcomes, as it was not possible to assess the impact on medium and long-term variables, such as exacerbations.

The 52 patients are certainly representative of a group of severe and very severe COPD treated by the SUS. The limitations do not preclude the main conclusions of this real-life evaluation of PCDT implementation. In a portion of patients, the recommendations resulted in a clear benefit. However, the majority of those who were switched to a supposedly more appropriate treatment did not notice a difference or felt harmed, even though they were members of an institution with a structured pharmaceutical care service. These, in particular, felt unassisted as they no longer had access to the medication with which they felt better. This distortion certainly requires consideration, review of implemented measures, and attention in the elaboration of future therapeutic guideline protocols, thinking not only about the regulation itself, but also about a health education process, through pharmaceutical assistance, simultaneously with the implementation of new therapies in order to minimize the impact on patients.

## Declaration of competing interest

The authors declare no conflicts of interest.
